# *In Vivo* 3D Histomorphometry Quantifies Bone Apposition and Skeletal Progenitor Cell Differentiation

**DOI:** 10.1038/s41598-018-23785-6

**Published:** 2018-04-03

**Authors:** Shu-Chi A. Yeh, Katarzyna Wilk, Charles P. Lin, Giuseppe Intini

**Affiliations:** 1 Department of Oral Medicine, Infection, and Immunity, Harvard School of Dental Medicine, Boston, MA 02115 USA; 2 Advanced Microscopy Program, Center for Systems Biology and Wellman Center for Photomedicine, Massachusetts General Hospital, Harvard Medical School, Boston, MA 02114 USA; 3 Harvard Stem Cell Institute, Cambridge, MA 02138 USA

## Abstract

Histomorphometry and Micro-CT are commonly used to assess bone remodeling and bone microarchitecture. These approaches typically require separate cohorts of animals to analyze 3D morphological changes and involve time-consuming immunohistochemistry preparation. Intravital Microscopy (IVM) in combination with mouse genetics may represent an attractive option to obtain bone architectural measurements while performing longitudinal monitoring of dynamic cellular processes *in vivo*. In this study we utilized two-photon, multicolor fluorescence IVM together with a lineage tracing reporter mouse model to image skeletal stem cells (SSCs) in their calvarial suture niche and analyze their differentiation fate after stimulation with an agonist of the canonical Wnt pathway (recombinant Wnt3a). Our *in vivo* histomorphometry analyses of bone formation, suture volume, and cellular dynamics showed that recombinant Wnt3a induces new bone formation, differentiation and incorporation of SSCs progeny into newly forming bone. IVM technology can therefore provide additional dynamic 3D information to the traditional static 2D histomorphometry.

## Introduction

Bone histomorphometry is commonly used to quantitatively assess changes in bone microarchitecture, bone morphogenesis, remodeling, and metabolism^1^. It offers the opportunity to perform a broad range of measures, including bone formation rate and quantification of cellular compositions; therefore, it has been an important tool in evaluating bone turnover^2^ under genetic manipulations^3^, presence of cancer^4^, or pharmacological intervention in bone metabolic diseases^5^. Histomorphometry relies on *ex vivo* post-mortem processing and typically involves time-consuming sample preservation and histological preparation, which potentially requires optimization trials to minimize distortion of microstructures^1^. In addition, as thin-sliced bones of 5 to 10 μm are usually used, the conventional approach inherently does not provide 3-dimensional parameters. Volumetric analysis of bone microarchitecture can be accomplished using micro-computed tomography (μCT) aided by finite element analysis^6,7^ and advanced reconstruction algorithms. Recent advances such as high-resolution *in vivo* μCT^8–10^ and high-resolution peripheral quantitative computed tomography (HR-pQCT)^11,12^ further allow time-lapse studies, with spatial resolution of tens of micrometers. However, radiation dose minimization remains an issue because of the potential impact of radiation on bone metabolism^13^. In addition, Micro-CT only provides morphological information, thus immunohistochemistry is still required to identify various cell populations.

Intravital microscopy (IVM) can overcome the limitations mentioned above^14,15^. Laser scanning confocal (LSCM) or multi-photon fluorescence microscopy (MPM) is capable of achieving optical sectioning with diffraction-limited spatial resolution, which renders 3D imaging of sub-cellular resolution after reconstruction^16^. This feature makes IVM a suitable tool for evaluating histology *in vivo*. For example, LSCM-based IVM has been used in clinical settings such as in dermatology^17^ and gastroenterology^18^. IVM, by acquiring real-time videos or time-lapse movies, can help visualizing and tracking biological processes at various time scales from milliseconds to days and weeks^14,19^. With advancement in transgenic mouse models^15,20^ and fluorescent probes to visualize cellular and molecular targets, IVM has been used as a powerful tool in examining dynamic cellular events, such as cell division^21^, migration^22,23^, and cell fate^24^ in the fields of immunology, stem cell biology, and bone research. For instance, IVM enables characterization of stem cell niche in the bone marrow cavities^14,25^, where expression of fluorescent proteins or exogenous fluorescent probe labeling at separable spectra allows for identification of various cells and cell lineages. Bone is readily identified by second harmonic generation (SHG) of type I collagen due to the organization of the collagen fibrils and the noncentrosymmetric molecular structures^26^. IVM has been used with fluorescent pH sensors^27^, or transgenic pH reporter mice^28^, to quantify local acidity and study bone resorption as well as osteoblast-osteoclast interactions^29^. More recently, IVM has also been employed to investigate the dynamic interactions between angiogenesis and osteoblast cells expressing *2*.*3Col1-GFP* over a 9-week time-course study of calvarial defect healing^30^.

The cranial suture has recently been identified as a stem cell niche for calvarial development^31–33^. Our recent finding using IVM revealed that calvarial skeletal stem cells expressing *Prx1 (Prx1* + *SSCs)*, a transcription factor essential in early limb bud development^34,35^, are located exclusively in the cranial sutures, and respond by up-regulating osteogenesis markers upon calvarial subperiosteal injection of mouse recombinant Wnt3a (rmWnt3a), an agonist of the canonical Wnt/β-catenin pathway^33^.

Canonical Wnt/β-catenin signaling has been shown to tightly regulate skeletal development and is well known to facilitate self-renewal of multi-potent mesenchymal stem cells (MSCs), direct the MSC cell fate towards osteoblastic lineage, enhance proliferation of osteoblast precursors, and inhibit adipogenesis, chondrogenesis, and osteoclastic bone resorption in a context dependent manner^36–38^. In calvarial development, genetic^39^ or pharmaceutical activation^40^ of Wnt signaling increases differentiation of adult osteoblasts and osteogenesis.

Here we describe an *in vivo* histomorphometric approach to evaluate the effects of the activation of Wnt signaling on calvarial bone formation and cell composition. To do so we examine the effect of canonical Wnt signaling on *Prx1* + *SSCs* and their progeny of the calvarial bone using two-photon IVM to simultaneously (i) visualize two different cell populations, (ii) track their cellular fate, and (iii) quantify cell number and dynamics of bone growth. We used a lineage tracing reporter mouse model based on the *Prx1-creER-EGFP;Rosa-loxP-STOP-loxP-tdTomato* mouse line^33^. In this mouse line, upon treatment with tamoxifen, the *CreER* recombinase excises a STOP cassette inducing overexpression of *tdTomato* fluorescent proteins in *Prx1* + cells and their progeny. We evaluated *Prx1* + cells and their progeny in the coronal suture for their contribution in bone formation when exogenous rmWnt3a is administered locally, by calvarial subperiosteal injections. Dynamic changes in terms of cell incorporation rate into the new bone, bone growth, population expansion, and suture volume are measured in live animals before and after administration of rmWnt3a.

## Results

### *In vivo*, multi-color two-photon imaging enables longitudinal tracking of cell differentiation and bone morphometry

**Figure 1:**
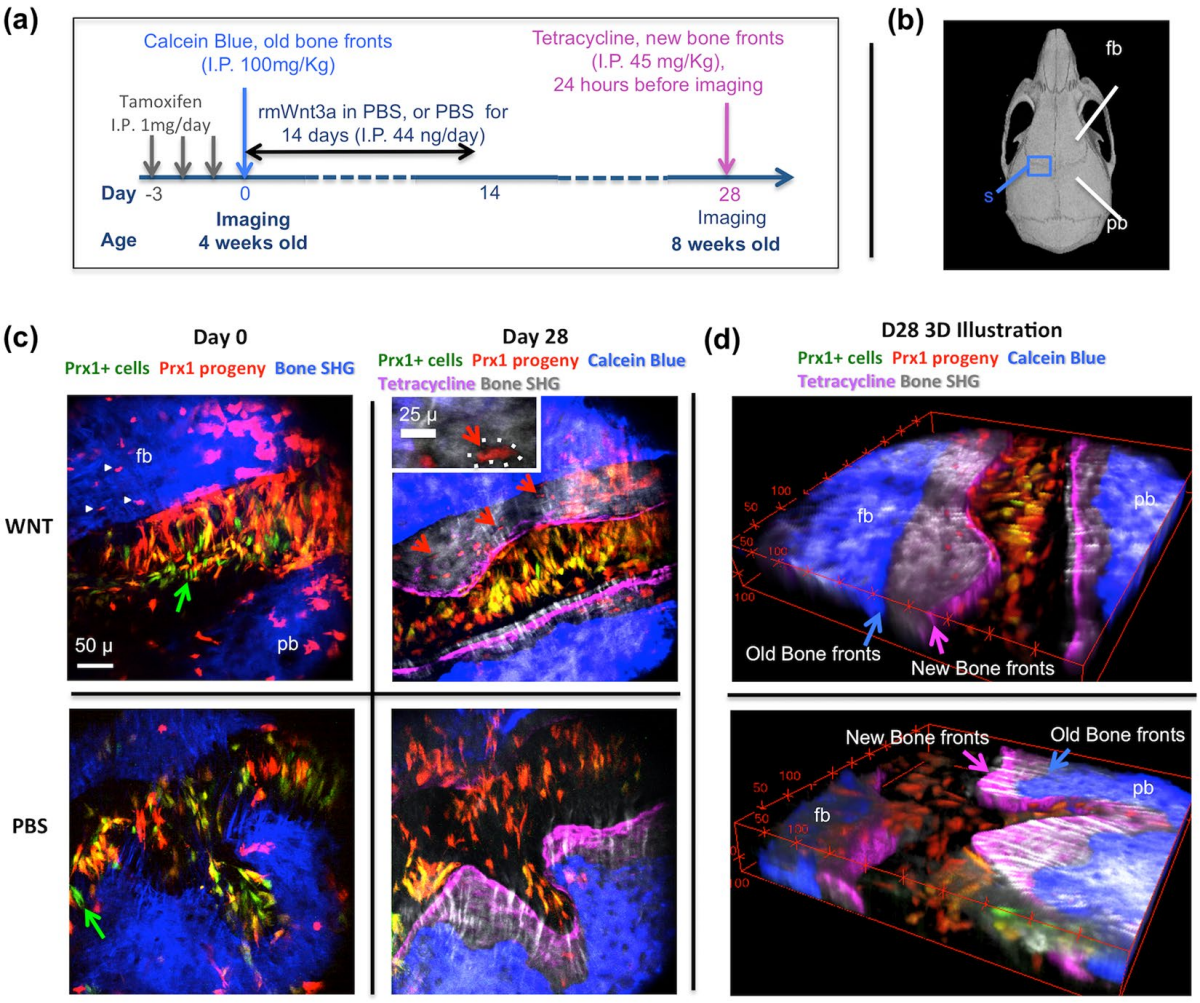
*In vivo*, multi-color two-photon imaging. **(a)** Injection regimens and imaging time points for *in vivo* histomorphometry. **(b)** The coronal suture space (S) between frontal (fb) and parietal (pb) bones. **(c)** Animals were treated with either rmWnt3a (in PBS, 44 ng/day) or just PBS for 14 days. Maximum intensity projection of a 25-μm layer was used for demonstration. Images on Day0 show *Prx1* + cells (Green), their progeny (Red or yellow, co-expression of *tdTomato* and *EGFP*). In addition, on Day 28, the initial bone structure was identified by calcein blue (Blue) and the new bone fronts were demarcated by tetracycline (purple), as indicated by blue and purple arrows in Fig. 1(d). Spontaneous recombination (white arrowheads) and incomplete recombination (green arrows) may be present. It is noted that a higher number of osteocytes originated from *Prx1* + cells were embedded in lacunae (the red arrows and the inset) from rmWnt3a treatment. **(d)** Quantifications of bone morphometry and cellular dynamics were performed over a 50-μm layer, as shown in 3D reconstructed images.

Lineage tracing reporter mice (*Prx1-creER-EGFP mice crossed into Rosa26LoxP*^*-STOP-loxP-tdtomato*^) were treated with Tamoxifen for 3 days to allow visualization of *Prx1* + cells and their progeny with *tdTomato* fluorescence (Fig. 1a**)**. Multi-color two-photon images of the coronal suture space (Fig. 1b, blue rectangle) were acquired at Day 0 and Day 28 using IVM before and after treating the animals with rmWnt3a or PBS for 14 days. Tetracycline and calcein, two calcium-binding fluorochromes that stain the bone mineralization fronts^41^, are administered intraperitoneally on Day 0 and Day 28, respectively, to define the old and new bone fronts. As shown in Fig. 1c, IVM revealed dynamic changes of bone structures visualized by SHG (Blue at Day0, Gray at Day28), old bone fronts stained by calcein blue (Blue, which corresponds to the SHG structure at Day0), new bone fronts labeled by tetracycline (Purple), the skeletal stem cells expressing *Prx1-creER-EGFP* (Green), their progeny expressing *tdTomato* fluorescence (Red or Yellow if co-expressing GFP), and the contribution of *Prx1* progeny to bone formation shown as *tdTomato* expressing (*tdTomato*+) osteocytes that reside in the lacunae^42,43^ in the newly formed bones (red arrows). As observed in Fig. 1c, a higher number of osteocytes were descendants of *Prx1* + cells in animals treated with rmWnt3a. IVM stack-images up to a depth of 50-μm enable quantification of histomorphometric parameters including cell number, bone volume, and suture volume (Fig. 1d).

Of note, we observed various extent of incomplete (*Prx1* + cells without *tdTomato* fluorescence) and spontaneous recombination (*tdTomato* + osteocytes present in the old bone) between regions and animals before treatment with rmWnt3a or PBS, as seen on Day 0 images in Fig. 1c. IVM therefore presents a major advantage that enables normalization of the measured parameters on D28 to the baseline values on Day 0, thus minimizing inter-regional and inter-subject variability during result interpretation.

### Image processing and quantitative analysis

**Figure 2:**
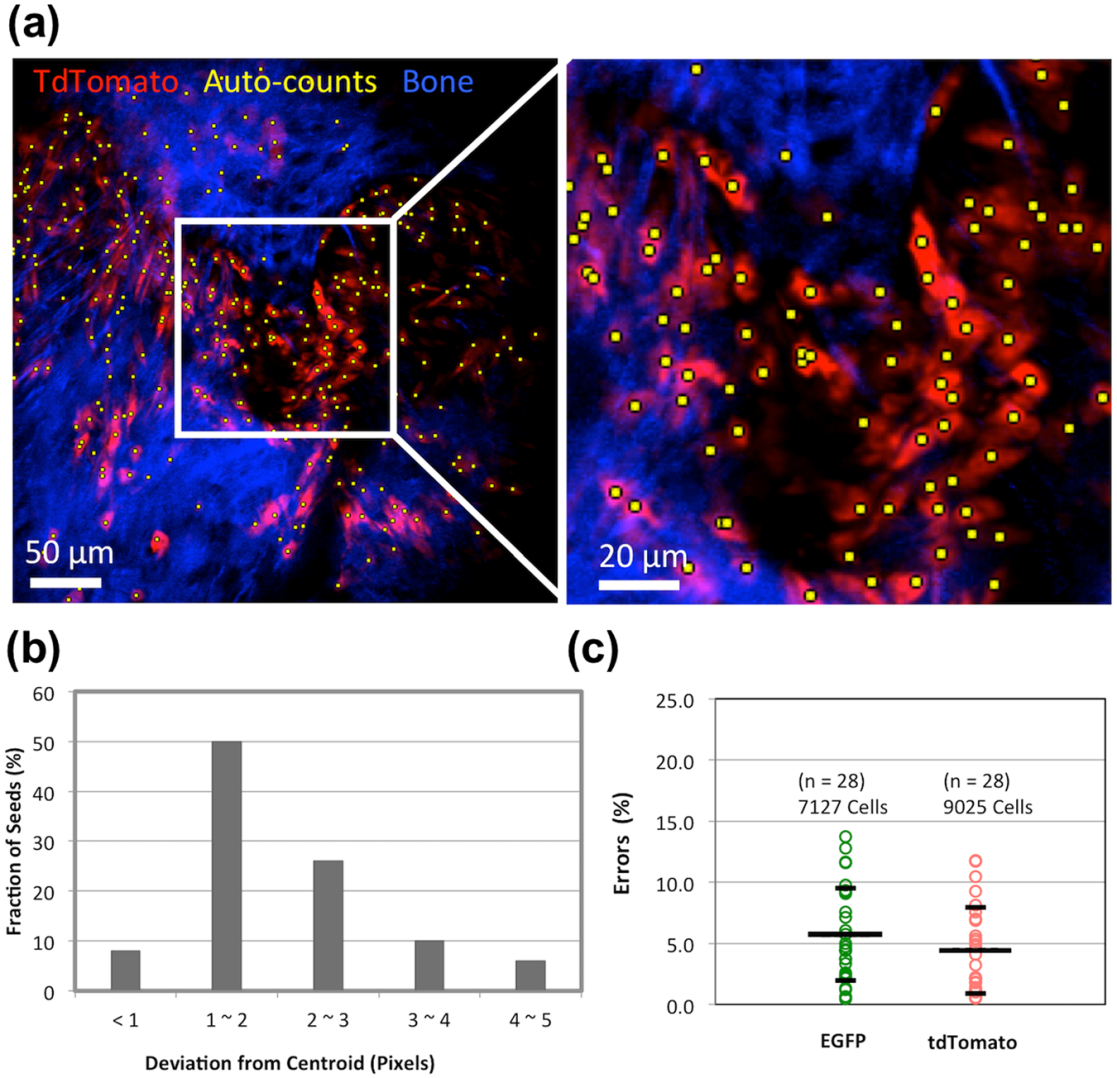
Image processing and quantitative analysis. Automatic cell counting adapted from Image J Plugins **(a)** Overlay of the identified seeds (yellow) with *tdTomato* + cells. Images were presented using maximum intensity projection. **(b)** Validation of automatic seed identification against 50 manually identified cells. **(c)** The counting accuracy of 5.7% ± 3.8% in the EGFP group, and 4.4% ± 3.5% in the tdTomato group was validated by 28 randomly picked regions (a total of 7127 and 9025 cells, respectively). Error bars represent standard deviation.

To quantify cell dynamics, image processing and automatic cell counting were performed to retrieve dynamic changes of each cell population. Figure 2a displays representative images showing “seeds” identified by automatic cell counting (yellow dots) overlaid on the *tdTomato* + cells (red). The identified seed reflects the 3D location of the strongest fluorescence emission found in each cell (Supplementary video 1). For 2D demonstration, the *tdTomato* + cells within the region of interest are displayed by maximum intensity projection (from 0 to 50 μm) and then superimposed with all the identified seeds. A zoomed region shows that the seeds identify the center of each cell in 3D, with a mean deviation of only 2 pixels in *xyz* coordinates when validated against the centroid of individual cells manually segmented then measured using *Image J (3D Object Counter\Centroid\Statistics)* **(**Fig. 2b).

To investigate the accuracy of automatically counted values, the results were compared with manual annotations of *Prx1* + and *tdTomato* + cells from 28 randomly picked regions, corresponding to a total of 7127 (*Prx1*) and 9025 (*tdTomato*) cells. In Fig. 2c, we show the counting accuracy between automatic counting and the ground truth was 5.7% ± 3.8% in the GFP group, and 4.4% ± 3.5% in the tdTomato group. We used standardized threshold adjustment based on Tukey’s anomaly (Methods and Supplementary Fig. 1), which performed better than global thresholding, enabling better identification of the seeds of dim cells (Supplementary Fig. 2). An algorithm that considers size variation of cells was also utilized to significantly improve the counting accuracy (Supplementary Fig. 3).

**Figure 3:**
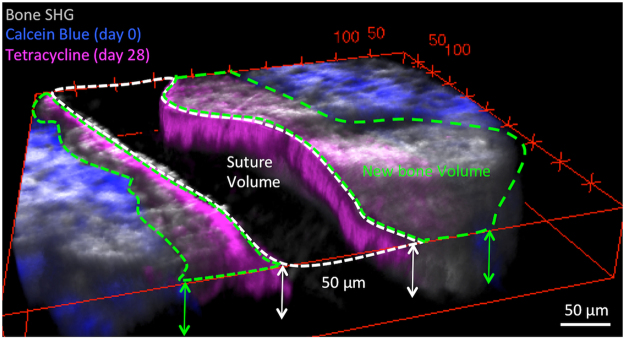
3D quantifications of bone morphometry. Volume measurement is demonstrated by a 3D reconstructed image. The bone fronts were manually segmented in each *z*-slice. The white dashed lines represent the suture area, and the green dashed lines represent the new bone area in the first slice. All the measured areas were summed over 50 μm in z dimension and converted to mm^3^.

To assess bone formation and bone volume, the bone fronts at Day 0 and Day 28 were manually segmented based on calcein blue and tetracycline margins, then the bound area between the bone fronts were calculated and summed over all the 3D slices to yield the volumes of the total newly formed bones, growth of frontal or parietal bones, and the coronal suture (Fig. 3). Automatic segmentation was not performed for volumetric analysis due to discrete staining pattern typically present by tetracycline labeling (Supplementary Fig. 4., indicated by purple arrows). In regions of missing tetracycline signal, the simultaneously acquired bone SHG signal has served to delineate the new bone fronts. Automated segmentation of the SHG images was hampered however by the bright collagen fibers in the suture space (Supplementary Fig. 4., indicated by white arrows), typically at perpendicular orientation to the bone fronts.

### Dynamic changes of bone histomorphometry in response to rmWnt3a

**Figure 4:**
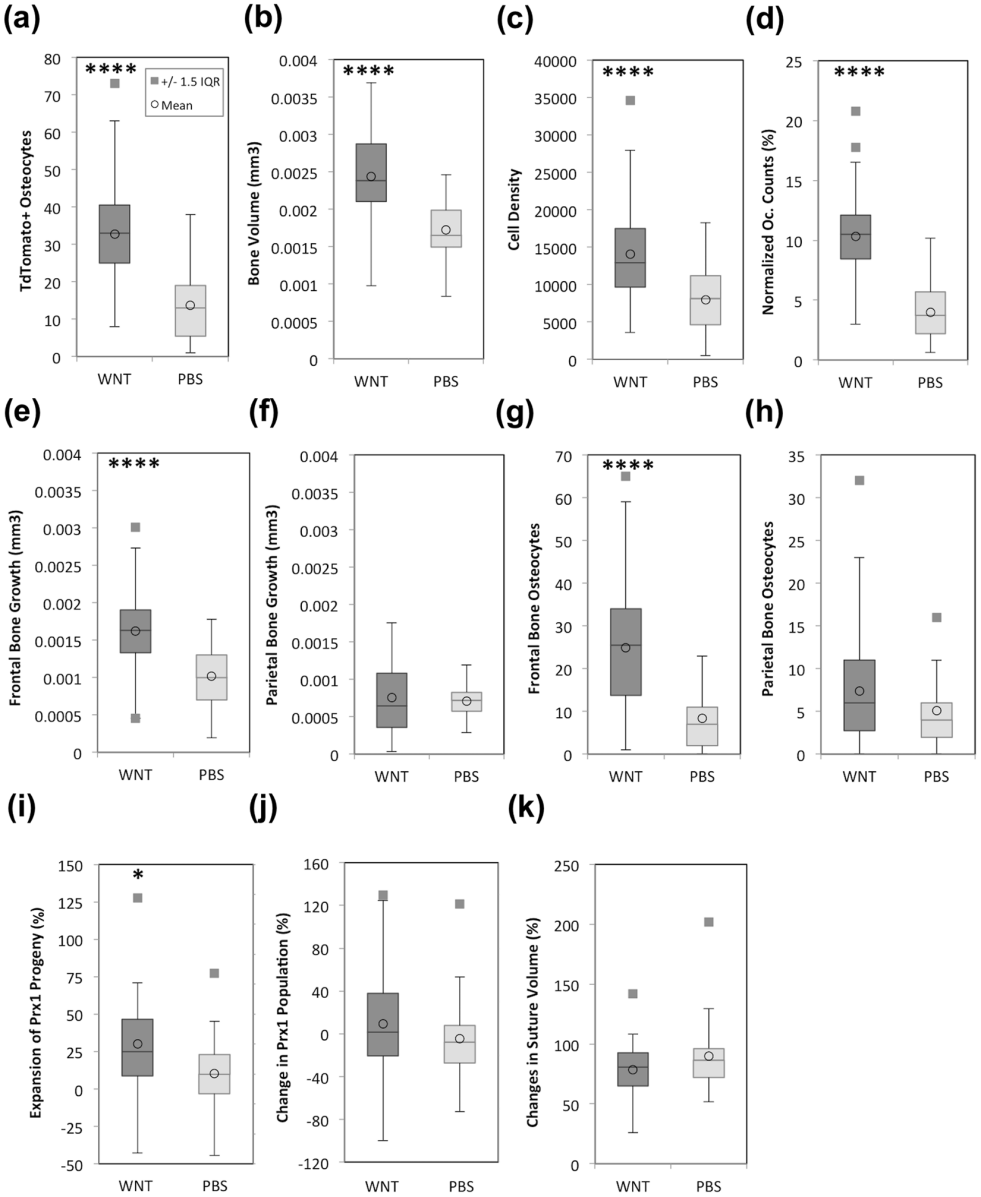
Dynamic changes of bone histomorphometry in response to rmWnt3a. Quantitative measurement of dynamic changes through a period of 28 days in response to rmWnt3a treatment compared to the control group. The box plots demonstrate the mean, median, the interquartile range, the whiskers showing the total range of the measurements, and the individual data points greater or less than by 1.5 times the interquartile range. **(a)** *tdTomato* + osteocytes derived from *Prx1* progeny **(b)** Bone growth **(c)** Cell density, *tdTomato* + osteocytes normalized to bone growth **(d)** *tdTomato* + osteocytes normalized to initial cell count at Day 0 **(e)** Frontal bone growth **(f)** Parietal bone growth **(g,h)** Osteocytes incorporated to frontal bone and parietal bone, respectively **(i)** Expansion of *Prx1* progeny **(j)** Changes of *Prx1* + population **(k)** Changes in suture volume. (N = 6 and 5 animals in rmWnt3a and PBS treated groups, respectively. 6 to 8 regions in the coronal suture of each animal were acquired).

It is observed in Fig. 1 that local delivery of rmWnt3a promotes incorporation of *Prx1* progeny during bone formation, as a higher number of *tdTomato* + osteocytes were found in the newly formed bone (red arrows, Fig. 1c). In Fig. 4, we processed the acquired intravital images to yield quantitative analysis. The box plots demonstrate the mean, median, the interquartile range, the whiskers showing the total range of the measurements, and the individual data points greater or less than by 1.5 times the interquartile range.

As shown in Fig. 4(a–c), compared to PBS treated animals, rmWnt3a treated animals showed an increased number of *Prx1* progeny as osteocytes (Fig. 4a) (33 ± 14 vs. 14 ± 9, p < 0.00005). In addition, 3-dimensional bone volume measurements through a 50-μm z-stack revealed that treatment with rmWnt3a increased bone apposition by 41% (p < 0.00005)(Fig. 4b). The increase in the number of *Prx1* progeny as osteocytes within the newly formed bone was not simply due to bone growth, as the cell density, calculated by normalizing the contribution of *Prx1* progeny to bone growth, also increased by 77% (P < 0.0005) compared to the control group (Fig. 4c). Note that the quantification is not affected by pre-existing *tdTomato* + osteocytes in the old bone, (Fig. 1c), since only the ones within the new bone volume **(**Fig. 3) were included in the analysis. By imaging the same animal at different time points, IVM further avoids bias that might arise from variations of baseline cell numbers before rmWnt3a treatment. Figure 4d presents the normalized cell counts of *tdTomato* + osteocytes to the initial *tdTomato* + cell number of the region, and shows a significantly higher fraction of *tdTomato* + cells that have committed cell fate to become osteocytes (10% ± 4% vs. 4% ± 2%, p < 0.00005).

Interestingly, and confirming the differential effects of agonist Wnt signals on the neuro-ectodermal derived frontal bone versus the mesoderm derived calvarial bone^44^, rmWnt3a had a differential effect on frontal and parietal bones: the former showed an increase of 59% (p < 0.00005) compared to the control group whereas the latter showed no significant increase (Fig. 4e–f). This discrepancy in bone growth correlated with the number of *Prx1* progeny as osteocytes (Fig. 4g,h, p < 0.00005). Incorporation rate of *tdTomato* + cells into osteocytes in frontal bones (*tdTomato* + Osteocytes/new frontal bone volume) in rmWnt3a treated animals was found to be 56% higher than the rate in parietal bones. In contrast, only 15% increase was found in the control group treated with PBS (data not shown).

The total number of *tdTomato* + cells, representing *Prx1* + skeletal stem cells and their progeny, was found to go through substantial expansion (30%) after rmWnt3a treatment in comparison with the PBS treated animals (10%). In contrast, no significant difference was observed when just comparing the *Prx1* + population (9% in the group given rmWnt3a as opposed to −5% in the control group) (Fig. 4i,j). In analyzing the expansion of *Prx1* progeny, all *tdTomato* + cells in the 3D volume were counted without excluding the pre-existing cells in the old bone, which were present in small numbers and did not significantly affect the outcome of the analysis. However, changes in population were normalized to the baseline cell number in order to take into account regional variations in cell numbers at Day 0. Lastly, treatment with rmWnt3a yielded little or no changes in suture volume at this age (Fig. 4k).

## Discussions

This work used two-photon, multicolor IVM as a novel histomorphometric tool to quantify changes in bone anatomical structures and track cell fate in 3D. We measured histomorphometric parameters through a 50 μm-thick bone layer using live animals, and obtained dynamic 3D volumetric analysis via image acquisition and processing at longitudinal time points. Bone morphology in 3D is visualized by SHG of collagen, with the old and new bone fronts demarcated by double staining with calcein blue (depicted in blue in the figures) and tetracycline (depicted in purples in the figures). *In vivo* tracking of cell function is further enabled by the lineage tracing reporter mouse model. Using IVM and image processing, we showed that local delivery of rmWnt3a promotes expansion of *Prx1* progeny and their incorporation in bone formation at the adolescent age, and that this effect is more prominent in the frontal bone compared to the parietal bone.

Canonical Wnt signaling has been extensively studied and known for facilitating osteogenesis or inhibiting endochondral bone formation in calvarial sutures. Its acts on specific skeletal cell population in a context dependent manner^3,37,40,45–47^. In the shown experiments, the quantitative results from IVM are consistent with the results of our previous study, where post-natal *Prx1* + cells were shown to go through osteo-differentiation when treated with rmWnt3a *in vitro* and *in vivo*^33^. Interestingly, adding exogenous rmWnt3a led to significantly more bone growth of the frontal bone, whereas such a change was not observed in the parietal bone compared to PBS treated group. Our results suggest that elevated canonical Wnt signaling may control predominantly morphogenesis of the anterior skull.

A number of factors may affect the quantifications. Mounting and stabilization of mouse calvaria is critical to obtain consistent field of view for cell counting between measurements. Additional attention should be paid to spectral separation between fluorochromes. For example, *Prx1-EGFP* fluorescence is much weaker than *tdTomato* fluorescence in this study; hence the detection gate of *EGFP* fluorescence was moved towards shorter wavelengths (480 nm–520 nm) instead of the typical green channel (500 nm–550 nm) to avoid leakage of *tdTomato* fluorescence into the *EGFP* channel. Careful selections of fluorochrome combinations for labeling bone fronts is also important to avoid spectral overlap with other cells of interest.

In Table 1, we summarize and compare pros and cons of our newly developed IVM 3D histomophometry with traditional *ex vivo* histomorphometry. Briefly, traditional histomorphometry is performed using separate cohorts of animals for analysis at longitudinal time points and lengthy sample preparation for histomorphometric analysis is also required. More importantly, by means of IVM, dynamic changes of bone and cellular events are acquired with minimal perturbation of natural physiological conditions. Although numerous advantages are present in the IVM approach, it should be noted that conventional histomorphometry possesses inherent flexibility in exploring multiple cellular/molecular targets of interest. For instance, fluorescence labeling of multiple cell populations can be achieved by immunohistochemistry, with fewer limitations in terms of the “types” of targets, as long as the associated biological markers can be identified and labeled. *Ex vivo* methodology with the help of a variety of fluorescent or chromogenic contrast agents can provide contrast between cartilages, mineralized bones, and osteoids. Similarly, IVM with additions of various modalities will allow studies of these structures. For example, SHG has been used in characterizing bone ultrastructure^48^, aging effects of cortical bones^49^, and the extracellular matrix^50^. IVM equipped with Raman scattering detection has allowed direct observation of mineralization processes^51,52^. In addition, many common chromogenic reagents are fluorescent^41,53^, such as Toluidine Blue for cartilage staining, and Alizarin Red utilized in discriminating mineralized bone and osteoid seam, and therefore they could become feasible for IVM 3D histomorphometry. Furthermore, IVM is also capable of imaging other non-cellular structures such as lipids (commonly stained with Oil Red in *ex vivo* histomorphometry) via the use of third harmonic generation (THG), Coherent Anti-stokes Raman Scattering (CARS) and development of optical biosensors^54–57^. In many occasions, *in vivo* immunohistochemistry can be achieved by local^33^ or systemic delivery of fluorescent antibodies^58,59^, given that specific surface receptors are present *in vivo*. In terms of tissue accessibility (i.e. tissue depth of the analyses), in conventional *ex vivo* histomophometry, a series of sections obtained throughout the specimens is commonly used. IVM has been shown to efficiently identify cell activity throughout various tissues and bone, such as in tibial bone^27,29^, in tibial bone marrow^60^, as well as in calvarial bones^61^. Signal attenuation of SHG in bone is more apparent due to its high scattering coefficient. The thickness of 50-μm used in this study ensured clear visualization of bone structures, and also standardized the measured suture areas as the coronal suture at this depth fits in one single field of view. Overall, the achievable imaging depth from two-photon excitation is typically from tens to hundreds of microns based on tissue optical properties^62,63^. Breakthrough in imaging depth will be an upmost advantage to allow thorough 3D analysis. For instance, the image depth can be further improved with 3-photon microscopy^64^. Last but not least, continuing advances in image processing of microscopic data, including the development of novel quantifying 3D algorithms^65^, would make IVM 3D histomophometry a promising tool to study bone metabolism and bone regeneration.

**Table 1:** Comparison of *ex vivo* and *in vivo* histomorphometry.

	Pros	Cons
IVM	Allowing longitudinal tracking of dynamic cell behavior. Simultaneous acquisition of histology and morphometric parameters. Well established in imaging calvaria. Also allows short-term tracking (within a week) of long bones. Retrieving 3D information is relatively more straightforward.	Relying on genetic mouse models and/or multi- modalities to probe targets of interest. Access to deep tissue is limited by penetration depth of light.
*Ex vivo*	Generating good contrast of mineralized bone and osteoids by chromogenic staining. Well established in imaging both long bones and calvaria. Flexibility in exploring a wide range of cell targets by using immunohistochemistry.	Requiring separate cohorts of animals for longitudinal histological studies although some morphometric parameters can be analyzed by micro-CT. Relatively more time-consuming in sample preparation.

## Conclusion

When tracking the cell fate and bone growth for a period of 28 days before and after rmWnt3a administration, IVM revealed an increased bone growth and an increased incorporation rate of osteolineage cells. Using customized cell counting algorithms, we showed rmWnt3a led to expansion of *Prx1* progeny, but not the *Prx1* + cells. We conclude that i*n vivo* IVM histomorphometry analysis allows for simultaneous visualization of histology and morphometric information and may be utilized to study the effects of anabolic or catabolic signaling during calvarial bone formation.

## Materials and Methods

### Animal models

All animal experiments were conducted in compliance with the institutional guidelines and approved by the Institutional Animal Care and Use Committee (IACUC) at Harvard Medical School (IACUC approval IS00000535) and Massachusetts General Hospital (IACUC approval 2015N000098). Lineage tracing was performed using *Prx1-creER-EGFP* mice crossed into *Rosa26LoxP*^*-STOP-loxP-tdtomato*^ (*tdTomato*) mice^33^. Both heterozygous and homozygous *Prx1-creER-EGFP* mice were used for this study. As homozygous *Prx1-creER-EGFP* mice may present more GFP cells, homozygous animals were divided evenly to Wnt and PBS groups. Also, since *Prx1* is expressed in ovary and the *creER* is sensitive to estrogen, to minimize spontaneous recombination only male mice were utilized throughout the studies.

### Reagents

10 mg/mL tamoxifen (T5648, Sigma, St. Louis, MO) was prepared in sterile oil. Calcein blue power (M1255, Sigma) was dissolved in 2% sodium bicarbonate solution to make a 3% working concentration. Mouse recombinant Wnt3a (1324-WN-002, R&D systems, Minneapolis, MN) was prepared at a concentration of 0.44 μg/mL in DPBS. 7.5 mg/mL tetracycline (T7660, Sigma) was prepared in sterilized water and NaCl was added to obtain isotonic solution immediately before injection.

### *In vivo* histomorphometry

As illustrated in Fig. 1, 4-week-old *Prx1-creER-EGFP;tdTomato* male mice were injected with tamoxifen (40 mg/kg in sterile oil, I.P.) for 3 consecutive days. Starting at Day 0, animals were treated with calvarial subperiosteal injections of either rmWnt3a (in PBS, 44ng/day) or the vehicle control (PBS) for 14 days. Calcein blue (100 mg/Kg, I.P.) was used to label the existing bone fronts on Day 0, and tetracycline (45 mg/Kg, I.P.) was administered 24 hours before the follow-up imaging (Day 28) to stain the new bone fronts.

### Intravital microscopy

IVM was performed as previously described at the time points Day 0 and Day 28^14^. In the IVM setup, the mouse head was stabilized in a mouse restrainer and maintained in anesthesia using 1.25% vaporized isofluorane (Fig. 2(a)). A skin flap was created without damaging the periosteum for *en face* acquisition of the coronal suture, the patent space between frontal (fp) and parietal bones (pb), as marked by the blue box in Fig. 2(b). Figure 2(c) shows 3D visualization of a sample region, containing the coronal suture and the *Prx1* + cells in a live animal. Specifically, the excitation beam was focused into the sample plane using a 60x objective lens (NA = 1), yielding a field of view of 400 × 400 μm. Fluorescence emission was then collected by photomultiplier tubes with proper dichroic and filter settings corresponding to fluorophores of interest. A Ti:Sapphire laser pulsing at 900 nm was used for acquiring SHG of collagen, fluorescence of *EGFP* and *tdTomato*, which was detected using 415–455 nm, 480–520 nm, and 605–650 nm band pass filters, respectively. The settings of the emission filters were intended to avoid signal crosstalks between *EGFP* and *tdTomato* fluorescence, which could be a confounding factor in cell counting. Using the same set of blue and green emission filters, 775 nm laser pulses were used to excite calcein blue (blue channel) and tetracycline (green channel). All image stacks were acquired at 1 μm interval starting from the surface of the skull. Image analysis was performed within the 50-μm layer, where the coronal suture was placed at the center of the field of view with no obvious tilt horizontally.

### Quantitative analysis

Multi-channel z-stack images were rendered and analyzed using Fiji^66^. Calcein blue emission leaked into the green detector was first subtracted before overlaying all emission channels. Osteocytes and mature osteoblasts expressing *tdTomato* were identified in the newly formed bone demarcated by calcein blue and tetracycline staining. When evaluating the incorporation of *Prx1* progeny in bone formation, *tdTomato* expressing (*tdTomato*+) mature osteoblast cells localizing right at the border lines marked by calcein blue and tetracycline were included, whereas cells appeared in the vessel walls, bone marrow cavities, and in the old bones were excluded. The incorporation rate was presented as “cell density”, calculated by the number of *tdTomato*+ osteocytes and osteoblasts divided by the new bone volume (shown in Fig. 4). The fractional changes of all the measured parameters (*ΔM*), including expansion of different cell populations and the suture volume, were calculated by:

$${\rm{\Delta }}M=({M}_{D28}-{M}_{D0})/{M}_{D0}\times 100$$

### Image processing for automatic cell counting

Automatic cell counting of *Prx1* + and *tdTomato* + cells in each stack was obtained using *Image J Macros* containing multiple Fiji plugins, including *Moment* segmentation, *3D image J suite*, and *3D object counter*. Specifically, cell areas were first segmented using the built-in segmentation algorithm “*Moment*” in Image J, followed by standardized contrast enhancement to allow 1.2% of saturated pixels in each stack. This step minimizes oversaturation, improves visibility of dim cells while not over-enhancing background intensity from autofluorescence or crosstalks between emission channels. Then, detection of the “seed” of each cell was achieved from a two-step process: (i) Using the *3D image J suite* plugins^67^, image stacks were processed with a combination of filters, including *3D Mean* (kernel size in all dimensions was set to 1 pixel), followed by *3D Maximum Local* to retrieve local maxima of individual cells. The kernel size in each dimension (*e*.*g*. x = 6; y = 8; z = 10 pixels) of the *3D Maximum Local* filter was determined based on estimated cell size. The x dimension is a particularly useful to separate cells located very close to each other (Supplementary Fig. 3). (ii) From the identified local maxima, *3D object counter*^68^ was applied as a second step to identify the centroid of each local maxima cluster and return the “seed”, which then represented the location of a single cell. It should be noted that a threshold was required in *3D object counter* to disregard “non-cell” local maxima clusters. To standardize threshold determination, we measured the outlier of “cell surrounding” (Supplementary Fig. 1a, shown in red) in each image, and the outlier was then considered as a true cell. Specifically, the seeds to be included (“the outliers” of cell surrounding) was determined based on *Tukey’s anomaly*^69^, as calculated in equations (1–3), where *Q1*, *Q3*, and *x* are the first quartile, third quartile, and the mean value obtained from the histogram of cell surroundings (Supplementary Fig. 1b). Eventually, *T*, the estimated outlier calculated in equation 3, was then used as a threshold for *3D object counter*. It should be noted that the image that had gone through more rigorous contrast enhancement (e.g. the max. intensity was only between 20 to 25 in a raw 8-bit image, Supplementary Fig. 1c) inherently yielded higher background intensity. To standardize segmentation of cell surrounding without taking excessive background noise, “step background gating” was applied. Specifically, the cut-off value at the lower bound of the histogram shown in Supplementary Fig. 1b was determined using the *mode* intensity (the background intensity) measured in each raw image, with step-adjustment according to the signal level (Supplementary Fig. 1c).

1 $$Q1=ln({4}/{3})/Exp(x)$$

2 $$Q3=ln({4})/Exp(x)$$

3 $$T=Q3+{1}.{5}\times ({Q3}-{Q1})$$

### Statistics

5 control (PBS) and 6 experimental (rmWnt3a) animals were used for the study. Six to eight regions in the coronal suture were acquired from each animal. Statistical analysis for mean and standard deviation was performed by pooling regions from all animals without excluding outliers. The plotted outlier was defined as a value less than or greater than 1.5 times the interquartile range. Statistical significant difference was obtained using two-tailed distribution equal variance Student’s t-test and results are shown as means ± S.D in figures unless specified otherwise. Statistical significant difference is indicated as *(p < 0.05), **(p < 0.01), ***(p < 0.005) or ****(p < 0.0005).

### Data availability

The datasets generated during the current study are available from the corresponding author on reasonable request.

## Electronic supplementary material

Supplementary Information

Supplementary Video 1.
