# Supplementary material for: In Vivo 3D Histomorphometry Quantifies Bone Apposition and Skeletal Progenitor Cell Differentiation
Source: Sci Rep. 2018 Apr 3;8:5580. doi: 10.1038/s41598-018-23785-6 (PMC5882859; doi:10.1038/s41598-018-23785-6)
Supplement: Supplementary file 1 — Supplementary Information [file 41598_2018_23785_MOESM1_ESM.pdf]

In Vivo 3D Histomorphometry Quantifies Bone Apposition and Skeletal Progenitor Cell Differentiation

Shu-Chi A. Yeh[1,2], Katarzyna Wilk[1], Charles P. Lin[2,3,*], Giuseppe Intini[1,3,*]

[1]*Department of Oral Medicine, Infection, and Immunity, Harvard School of Dental Medicine, Boston, MA, 02115, USA*
[2]*Advanced Microscopy Program, Center for Systems Biology and Wellman Center for Photomedicine, Massachusetts General Hospital, Harvard Medical School, Boston, MA, 02114, USA*
[3]*Harvard Stem Cell Institute, Cambridge, MA, 02138 USA*
**Co-corresponding authors*
Contact Information
Email: lin@helix.mgh.harvard.edu
Email: Giuseppe_Intini@hsdm.harvard.edu;

Supplementary Information

Supplementary Fig. 1. Step background gating

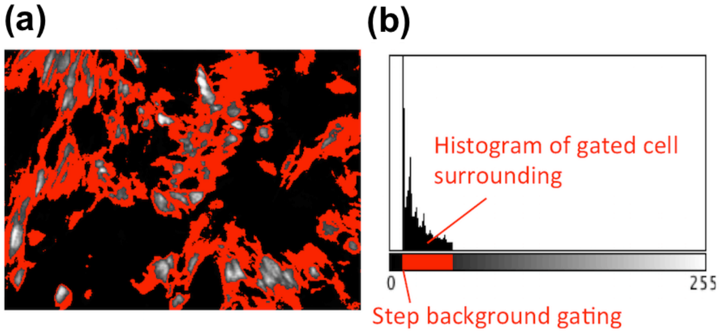

(c)

Intensity Level (8-bit)	Step Background gating
20 - 25	Mode + 8
26 - 29	Mode + 3
30 - 39	Mode
40 - 49	Mode - 2
50 - 74	Mode - 3
75 - 99	Mode - 4
100 - 124	Mode - 5
125+	Mode - 6

S1. **(a**) Cell surrounding, indicated in Red, and **(b)** its histogram. **(c)** Step background gating was used to determine the lower bound of the histogram.

Supplementary Fig. 2. Improved counting accuracy in step background gating

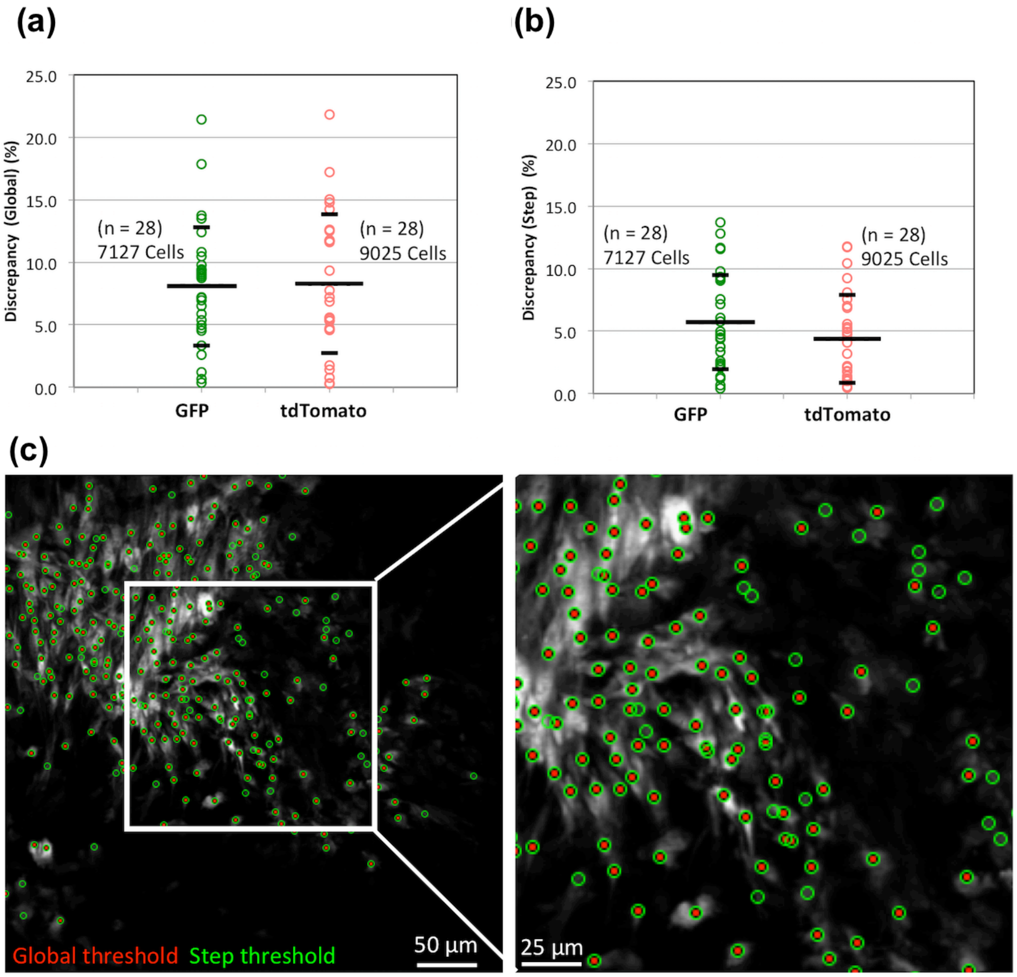

S2. **(a-b)** Step background gating (**suppl Fig. 1c**) improved the counting accuracy, as compared to over 8% error when using a global value over all images. Error bars represents standard deviation. **(c)** Dim cells can be identified using step threshold (Green) compared to global threshold (Red).

Supplementary Fig. 3. Cell size variability

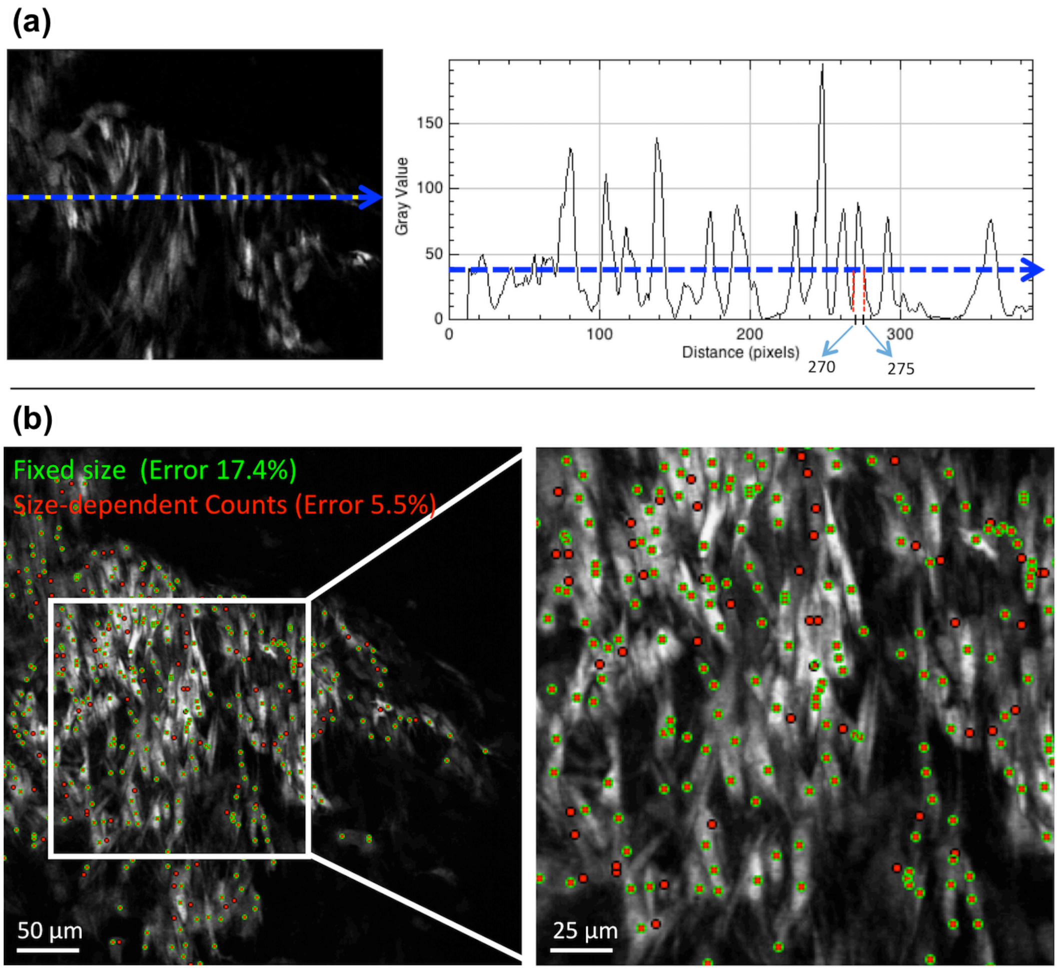

S3. Using the *3D image J suite* plugin, image stacks were processed with a combination of filters, including *3D Mean* (kernel size in all dimensions was set to 1 pixel), followed by *3D Maximum Local* to retrieve local maxima of individual cells. The kernel size in x dimension of the *3D Maximum Local* filter is varied based on estimated cell width: **(a)** Cell width can be estimated based on full width half maximum of clear "peaks" from the intensity distribution across the blue line (indicating an individual cell body). **(b)** The Size-dependent measurement (Red dots) better distinguished the cells closely adjacent to each other compared to the measurement using a fixed value.

Supplementary Fig. 4. Image contrast of new bone formation

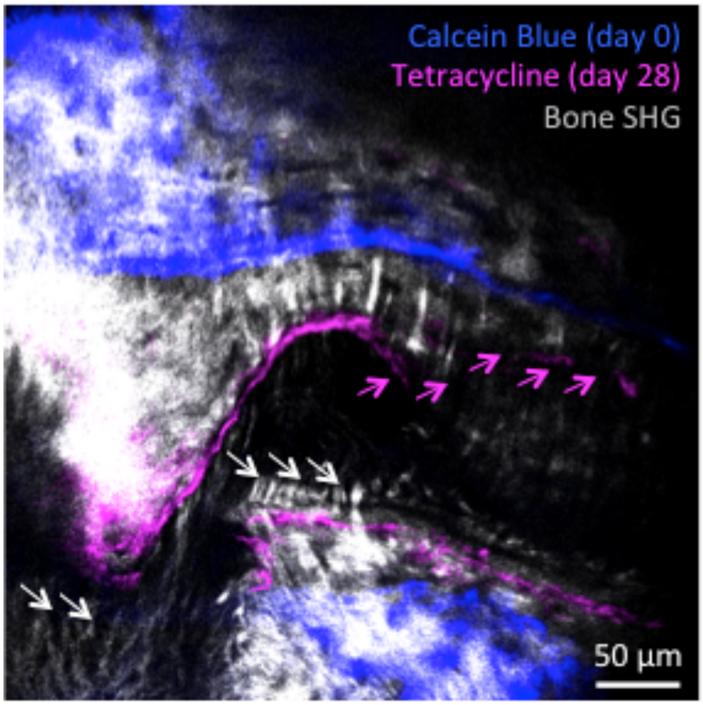

S4. 2D image at each z-plane is used for demarcating new bone and suture areas through the z-stack. The image provides a fair clear fluorescence lines and bone structure (via SHG) for manual annotation of bone fronts, whereas requires interactive user input for automatic segmentation. Purple arrows indicate the discontinuous staining lines from tetracycline. White arrows indicate SHG from collagen fibers present in the suture space.

Supplementary Video 1. Automatic cell counting to identify the seed of each cell in a 3D volume (uploaded separately)
